# Effect of Fermented Soy Beverage on Equol Production by Fecal Microbiota

**DOI:** 10.3390/foods13172758

**Published:** 2024-08-29

**Authors:** Ana Ruiz de la Bastida, Susana Langa, José Antonio Curiel, Ángela Peirotén, José María Landete

**Affiliations:** Departamento de Tecnología de Alimentos, Instituto Nacional de Investigación y Tecnología Agraria y Alimentaria (INIA-CSIC), Carretera de La Coruña Km 7.5, 28040 Madrid, Spain; ana.ruiz@inia.csic.es (A.R.d.l.B.); langa.susana@inia.csic.es (S.L.); angela.peiroten@inia.csic.es (Á.P.); landete.josem@inia.csic.es (J.M.L.)

**Keywords:** equol, fermented soy beverage, isoflavones, fecal microbiota

## Abstract

Soy consumption is associated with health benefits, mainly linked to the ability of the intestinal microbiota to metabolize the glycosylated isoflavones into more bioactive compounds, such as equol. Because *Bifidobacterium pseudocatenulatum* INIA P815 is able to efficiently deglycosylate daidzin into daidzein, the aim of this work was to confirm the influence of soy beverages fermented by *B. pseudocatenulatum* INIA P815 for enhancing equol production by fecal microbiota. Firstly, fecal samples from 17 participants were characterized in vitro, and we observed that 35.3% of them were able to produce equol from daidzein. In addition, the kinetics of equol production and degradation by fecal microbiota were evaluated, determining that 30–85% of equol is degraded after 24 h of incubation. Finally, the influence of fermented soy beverage on improving the production of equol by selected equol-producing fecal samples and by the equol-producing strain *Slackia isoflavoniconvertens* was analyzed through a colonic model. Fermented soy beverage enhanced the equol production from *S. isoflavoniconvertens* as well as the fecal samples whose microbiota showed high rates of equol degradation. The results obtained confirm that the fermentation of soy beverages with selected bacterial strains improves the functional properties of these beverages in terms of isoflavone metabolism and equol production.

## 1. Introduction

Soy intake is epidemiologically associated with beneficial health effects, mainly for menopause, cardiovascular diseases and metabolic syndrome, although beneficial effects for some types of cancer and neurodegenerative diseases have also been observed [1,2,3,4,5]. However, interventional studies aimed at elucidating the effects of soy isoflavone consumption in menopausal women were not as conclusive because of insufficient evidence and the contradictory results of collected studies as the European Food Safety Authority (EFSA) revealed in 2012 in a systematic review [6]. The inconsistency of those results found by the EFSA may be due to the fact that the positive effects of soy are mainly evidenced in the health of individuals whose intestinal microbiota metabolizes isoflavones producing equol [7]. The isoflavones present in soy foods are found mainly in their glycosylated and/or methylated forms, which have poor bioavailability and bioactivity, so they need to be metabolized by the intestinal microbiota to be absorbed into the intestine and exert their beneficial effects [7,8,9]. Their transformation by intestinal microbiota into the aglycone daidzein is necessary for equol production via the intermediary compounds dihydrodaidzein (DHD) and tetrahydrodaidzein (THD) [10]. Individuals able to produce equol should exhibit the microorganisms that carry out the reactions for its formation in their intestines. Thus, the human population can be divided into equol-producers (EP) and non-equol producers (NEP) [7]. The former would have an additional beneficial effect from soy consumption as a consequence of the production of equol and its intermediate compounds by their intestinal microbiota, while NEP individuals would only have the beneficial effect of compounds with fewer physiological effects compared to equol, such as daidzein, genistein, *O*-desmethylangolesin (*O*-DMA) or 6-hydroxy-*O*-desmethylangolensin (6-OH-*O*-DMA). According to this classification, between 50 and 60% of the Asian population is considered EP, while only between 20 and 35% of the Western population can produce this compound, albeit mainly in low concentrations [7,11].

Almost all the EP microorganisms identified to date belong to the *Eggerthellaceae* family, and EP strains have been isolated from the species *Adlercreutzia equolifaciens*, *Assacharobacter celatus*, *Enterorhabdus mucosicola*, *Slackia isoflavoniconvertens* and *Slackia equolifaciens* [10]. The genes *dzr*, *ddr*, *tdr* and *ifcA* are involved in producing equol and are found in the same operon, which maintains a similar organization in the different strains [8,12].

The principal benefits associated with equol are estrogenic properties, due to its ability to bind to estrogen receptors, and its antioxidant properties [13]. Furthermore, because equol exhibits higher bioavailability and slower clearance, their pharmacokinetics are different from those of daidzein [14,15]. Vasomotor disorders associated with perimenopause and menopause, such as sweating and hot flashes produced by the decrease in estrogen, were significantly reduced by supplementation with equol in NEP individuals [16]. Equol supplementation reduces bone resorption, increases bone mass density in both EP and NEP women [17,18] and improves skin health [19,20]. In addition, the effects of equol in the prevention of cardiovascular diseases, metabolic syndrome, reduction in the incidence of certain cancers and the prevention of dementia have been demonstrated [7,21,22,23].

Previously, we demonstrated the ability of *B. pseudocatenulatum* INIA P815 to efficiently increase the daidzein concentration during soy beverage fermentation through its ability to deglycosylate both free and bound daidzin to proteins and other substrates [24]. Therefore, our objective in this work was to assess whether a soy beverage enriched in daidzein by fermentation with *B. pseudocatenulatum* INIA P815 could enhance the production of equol by the fecal microbiota of EP individuals. To carry out our objective, the selection of EP individuals and the kinetic studies of equol production by their fecal microbiotas were performed, comparing the effect of the fermented soy beverage (FSB) with a control soy beverage (SB). Moreover, the influence of the SB and FSB on the production of equol by *S. isoflavoniconvertens* DSM 22006 and the degradation of equol by the intestinal microbiota were also addressed in this work.

## 2. Materials and Methods

### 2.1. Collection of Fecal Samples

Fecal samples were donated by 17 healthy volunteers (eleven women and six men) aged 22–67 years old. Fecal samples from eight participants were collected in February 2023, while samples from the remaining nine volunteers, who had already participated in a previous study to analyze equol production by their intestinal microbiota, were collected in March 2015 [25]. None of the participants used antibiotics in the three months previous to the sample collection, and they all followed a non-specified Western diet. The female volunteers were neither pregnant nor lactating. The volunteers were informed of the study objectives and gave their written consent. All the procedures involving human participants were in accordance with the Helsinki Declaration (1964) as well as its later amendments or comparable ethical standards, and the protocol was approved by the ethics committee of INIA (permission SG/RRHH-LCH).

Participants were provided with a sterile tray, a jar with a screw-top lid, a biohazard bag, and a wooden stick with an explanatory guide of the collection method. Briefly, participants had to place the collection targets in the bathroom where they could easily reach them. After washing their hands, they had to place the collection tray on the edge of the toilet bowl, in order to defecate on it without urinating. With the wooden stick, they collected the feces in the jar until they filled it to the indicated mark. Once collected, they placed the jar inside the biohazard bag, discarded the collection tray and the wooden stick, and washed their hands again.

### 2.2. Identification of EP Fecal Microbiota

Fecal samples were processed by means of homogenization in 24 h-reduced phosphate-buffered saline (PBS) 1× (20 g in 100 mL) followed by 2 min homogenization using a Stomacher (Seward; Worthing, UK). Subsequently, 500 µL of the processed feces were added to 4.5 mL of 24 h-reduced Wilkins-Chalgren anaerobe broth (WCAB) (Oxoid Ltd., Basingstoke, Hampshire, UK) containing a final concentration of 10 mg/L of daidzein (Merck; Madrid, Spain). The fecal suspensions were incubated by shaking under anaerobic conditions (10% H_2_, 10% CO_2_ and 80% N_2_. Whitley DG250 Anaerobic workstation, Don Whitley Scientific Limited, West Yorkshire, UK) at 37 °C for 48 h. WCAB with daidzein without fecal suspension was used as a control and incubated under the same conditions. Fecal suspensions were removed by centrifugation at 1372× *g* for 15 min. Afterwards, isoflavones from 5 mL of supernatants were extracted, once with 2 mL of diethyl ether and twice with 2 mL of ethyl acetate. The solvents were evaporated, and the residue was dissolved in 500 µL of methanol/water (50:50, *v*/*v*) and filtered through a 0.22 μM cellulose acetate filter (Millipore, Madrid, Spain). After centrifugation of 21,952× *g* for 10 min, the supernatants were transferred into HPLC vials and stored at −20 °C until the determination of equol production by HPLC-ESI-MS [26]. Briefly, the mass spectra of the extracted samples were obtained using a HPLC-PAD Beckman System Gold (Beckman Coulter Inc., Fullerton, CA, USA) coupled to a LC-MS Agilent 1200 (Palo Alto, CA, USA). Separation of compounds was achieved on a reverse phase Nova-Pak C18 column (300 mm × 3.9 mm, 4 μm) (Waters, Barcelona, Spain). The range acquisition 100–1000 *m*/*z*, gas temperature 350 °C, gas flow 10 L/min, nebulizer 45 psig, sheath gas temperature 350 °C, sheath gas flow 11 L/min, capillary voltage 3500 V, and fragmentation voltage 120 V were selected for the ESI/MS detection. The mass spectrometer operated in the negative ion mode. Other compounds derived from the daidzein metabolism such as DHD and *O*-DMA were also analyzed.

### 2.3. Identification of the Tetrahydrodaidzein Reductase (tdr) Gene in Fecal Samples

The molecular detection of the *tdr* gene, key to converting THD into equol, was carried out by PCR using the primers F-tdr 5′-CATTTCCCCACCAAGCA (G/A) GAGGGC and R-tdr 5′-GACATCTTCACCGAGAC (T/C) CCGGCCA, both based on the sequences of the *tdr* genes from *S. isoflavoniconvertens* DSM 22006 and *Adlercreutzia equolifaciens* DMS19450. The DNA was extracted from the fecal samples by means of the QIAamp PowerFecal Pro DNA KIT (Quiagen, Hilden, Germany) and DNA AmpliTools Master Mix (Biotools B&M Labs, S.A., Madrid, Spain) was used for the amplification reactions. Amplicons of approximately 320 nucleotides were purified by using QIAquick Gel Extraction Kit (Qiagen, Hilden, Germany) and sequenced at Secugen S.L. (Madrid, Spain).

### 2.4. Kinetics of Equol Production by the Fecal Microbiota

In order to evaluate the dynamic production of equol, four randomly selected EP fecal samples were incubated by shaking in 24 h-reduced WCAB supplemented with daidzein (10 mg/L). The concentrations of equol produced were monitored at different time points (24, 48 and 72 h) by collecting aliquots of each sample. Finally, the polyphenols were extracted and analyzed following the procedure described above [26].

### 2.5. Study of Equol Degradation by Fecal Microbiota

Fecal samples from five EP and five NEP were incubated at 37 °C with shaking under anaerobic conditions (10% H_2_, 10% CO_2_ and 80% N_2_. Whitley DG250 Anaerobic workstation, Don Whitley Scientific Limited, West Yorkshire, UK) in 24 h-reduced WCAB supplemented with equol (10 mg/L). After 24 h of incubation, the polyphenols of each sample were extracted in order to determine the residual concentration of equol by HPLC-ESI-MS [26]. In addition, other isoflavones such as 5-hydroxy-equol and probable phenolic compounds derived as a consequence of the microbial metabolism (Table 1) were also analyzed.

### 2.6. B. pseudocatenulatum and S. isoflavoniconvertens Culture Conditions

*B. pseudocatenulatum* INIA P815 isolated from human feces [27] (Genbank accession number of 16S rRNA sequence: MH114979.1) was grown in MRS broth (BD; Becton, Dickinson & Co., Le Pont de Claix, France) supplemented with 0.5 g/L L-cysteine (Merck KGaA, Darmstadt, Germany) at 37 °C under anaerobic conditions (10% H_2_, 10% CO_2_ and 80% N_2_. Whitley DG250 Anaerobic workstation, Don Whitley Scientific Ltd., Shipley, UK). *S. isoflavoniconvertens* DSM 22006 isolated from human feces (Genbank accession number of 16S rRNA sequence EU286403; and whole genome shotgun sequence, QIBZ00000000) was grown in plate in Agar Wilkins-Chalgren (Oxoid Ltd., Basingstoke, Hampshire, UK) at 37 °C under anaerobic conditions (10% H_2_, 10% CO_2_ and 80% N_2_. Whitley DG250 Anaerobic workstation, Don Whitley Scientific Ltd., Shipley, UK).

### 2.7. Soy Beverages Preparation and Isoflavone Analysis

SB consisted of the commercial soy beverage VegeDía (15% soy) (DIA, Madrid, Spain). FSB was prepared through the fermentation of the same commercial soy beverage by *B. pseudocatenulatum* INIA P815 according to Ruiz de la Bastida et al. (2023) [28]. Isoflavones from both SB and FSB were extracted. Briefly, 1 mL of beverage was extracted with 500 µL of acetonitrile, mixing by rotation for 1 h, and the extract was subsequently obtained by centrifugation at 21,952× *g* for 10 min. The extracts were filtered through a 0.22 μm cellulose acetate filter (Millipore, Madrid, Spain) and analyzed using HPLC-PAD (Beckman System Gold, Beckman Coulter Inc., Fullerton, CA, USA) according to Gaya et al. (2016) [26].

### 2.8. Effect of Soy Beverages on Equol Production by S. isoflavoniconvertens DSM 22006 in a Colonic Model

The equol production by the EP strain *S. isoflavoniconvertens* DSM 22006 was evaluated in a colonic model with three different daidzein sources: (1) pure daidzein in PBS 1× (5 mg/L (19.67 µM) final concentration in the fermenter), (2) SB and (3) FSB. The colonic model was based on an in vitro fermentation system previously described by Vulevic, Rastall and Gibson (2008) [29] and modified by Landete et al. (2020) [30]. The fermentation medium was previously prepared with the following composition: peptone water 2 g/L (Oxoid; Madrid, Spain), yeast extract 2 g/L (Oxoid), NaCl 0.1 g/L, K_2_HPO_4_.3H_2_O 0.05 g/L, KH_2_PO_4_ 0.04 g/L, MgSO_4_.H_2_O 0.005 g/L, CaCl_2_ 0.005 g/L, NaHCO_3_ 2 g/L, Ox-gal bile salts 0.5 g/L (Fisher; Madrid, Spain), Cys-HCl 0.5 g/L, Tween 80 ACROS Organics TM 2 mL/L, Hemin 0.02 g/L (Sigma; Madrid, Spain), Glucose (1%), Vitamin K 10 µL/L, pH 7.0. Each fermenter contained 150 mL of the fermentation medium, 19 mL of PBS 10X, 1 mL of *S. isoflavoniconvertens* inoculum and 30 mL of any of the mentioned daidzein sources. The fermenters were incubated anaerobically at 37 °C with constant shaking for 48 h. Samples were taken from each fermenter at 0, 6, 12, 24 and 48 h. The pH of the fermenters was maintained at 7.0 throughout the incubation. The samples were preserved at −80 °C until the moment of extraction and analysis. The samples were extracted using the ethyl acetate-diethyl ether method described above and analyzed with HPLC-PAD (Beckman System Gold, Beckman Coulter Inc., Fullerton, CA, USA) according to Gaya et al. (2016) [26].

### 2.9. Effect of Soy Beverages on Equol Production by Fecal Microbiota in a Colonic Model

The two EP fecal samples that produced the highest concentrations of equol after 24 h in the WCAB medium were selected to evaluate their production of equol in the presence of the SB and FSB beverages following the in vitro colonic model, using one NEP fecal sample as the control. Each fermenter contained 160 mL of fermentation medium, 30 mL of the corresponding beverage (SB or FSB) and 10 mL of the fecal solution of the corresponding donor in PBS 1× (final dilution 1:100 in the fermenter). The fermenters were incubated anaerobically at 37 °C with constant shaking for 48 h. Samples were taken from each fermenter at the following times: 12, 24 and 48 h, and their pHs were adjusted to 7.0 when required. Samples were kept at −80 °C until their extraction and analysis. The samples were extracted using the ethyl acetate diethyl ether method described above and analyzed with HPLC-PAD (Beckman System Gold, Beckman Coulter Inc. Fullerton, CA, USA) according to Gaya et al. (2016) [26].

### 2.10. Statistical Analysis

Fermentations were carried out in triplicate for each analysis. Statistical analysis of the data was performed using SPSS, version 22.0 (IBM Corp., Armonk, NY, USA). Data were analyzed by analysis of variance using a general linear model. Comparison of means was carried out using Tukey’s test.

## 3. Results and Discussion

### 3.1. Selection of EP Fecal Microbiota

The production of equol by the fecal microbiota of 17 individuals was analyzed. As is known, the human population can be classified into EP and NEP phenotypes based on the gut microbiota metabolism of daidzin. Although only 25–50% of the human population is considered EP, it is notable that 80–90% of NEP can produce other secondary daidzein compounds, such as *O*-DMA [10]. Of the 17 fecal samples, three out of six males and three out of eleven females were EP, resulting in 35.3% of EP in the group tested (Table 2).

In a previous study, we carried out a trial where the metabolism of phytoestrogens from the fecal microbiota of 14 adults was analyzed, and only one of the individuals showed equol production, resulting in 7.1% of EP in that group tested [25]. The increase in the number of EP individuals in this work may be because of the use of daidzein as an equol precursor, while in the previous one, only soy extracts were used, which mainly contained daidzin, the glycosylated form of daidzein as a potential precursor of equol [25].

Nine of the individuals who participated in the previous study [25] were also included in our trial. Among them, three showed a change in their EP phenotype. The individual FS1, categorized as EP in the previous study, did not show equol production in this intervention, while two previous NEP fecal samples changed to EP phenotype in this work (FS12 and FS13). Although the presence of daidzein facilitates equol production, FS1 did not show equol production. Hence, although the EP phenotype has been described as a relatively stable phenomenon [31,32], changes in the EP status could occur with similar frequencies as those observed in this work, especially when including testing periods superior to one year [31,33,34]. Moreover, there are other factors, such as antibiotic consumption throughout life, or changes in diet or lifestyle which can influence the phenotypic and genotypic changes of the intestinal microbiota [34].

The production of other compounds such as DHD and *O*-DMA was also analyzed. DHD production was observed in all the samples analyzed (data not shown), whereas the fecal samples from most of the donors were able to produce *O*-DMA (Table 2), agreeing with Frankenfeld et al. (2005) [33]. *O*-DMA was produced by 100% of the EP and 81.8% of the NEP (Table 2).

Finally, the presence of *tdr* in the fecal samples, which encodes the THD reductase [8,35], was analyzed, demonstrating that 88% of the tested individuals showed the presence of the gene responsible for the transformation of THD into equol in their fecal samples, independently of their EP status (Table 2). The sequencing of the amplicons obtained by PCR confirmed that they corresponded to the *tdr* gene described in *S. isoflavoniconvertens* or *A. equolifaciens*. Although this gene has been detected in EP fecal samples, the proportion of positive results of NEP observed in our study was higher than previously described [36,37]. The appropriate design of degenerate primers could result in more efficient molecular detection of EP fecal samples since detection of *tdr* may not be a good indicator of EP status. Our results suggest the presence of equol-related species of microorganisms in the majority of the individuals evaluated, although that did not translate into equol production according to observations from other studies [38,39]. Therefore, the NEP phenotype of some individuals may not be due to the mere lack of a specific group of EP bacteria but to the lack of an effective transformation of daidzein into equol due to underlying factors [39].

### 3.2. Kinetics Production and Degradation of Equol by Fecal Microbiota

**Figure 1:**
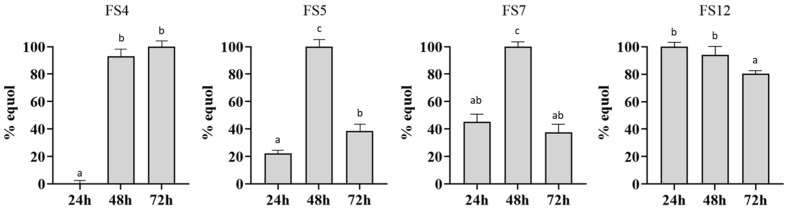
Kinetics of equol production and degradation by FS4, FS5, FS7 and FS12 fecal samples (FS). Values are expressed as percentage considering 100% of the maximum concentration of equol produced by the microbiota contained in each fecal sample. Values are mean ± SD. Different letters (a–c) indicate statistically significant differences (*p* < 0.05) by Tukey’s test.

Once the fecal samples were characterized, the EP fecal samples FS4 and FS5 from women, as well as FS7 and FS12 from men, were randomly selected and incubated in the presence of daidzein with the objective of quantifying their dynamic production and consumption of equol after 24, 48 and 72 h (Figure 1).

The kinetics of equol production and degradation of each fecal sample were different and therefore dependent on the microbiota present in each sample. Regarding this, except for FS4, all the fecal samples exhibited equol after 24 h of incubation in WCAB supplemented with daidzein, FS12 being the only fecal sample that reached its highest concentration at this point. After 48 h, all the samples considerably increased their equol concentration, reaching maximum levels for FS5 and FS7, while FS12 showed a slight decrease. Finally, at 72 h of incubation, all the samples showed a decrease in their equol concentrations, this degradation being very marked in FS5 and FS7, while FS4 showed its maximum concentration.

Despite the detection of equol, the compounds derived from microbial degradation proposed in this study (Table 1), which have been described in the biotransformation of flavonoids [40,41], were not found.

On the other hand, with the aim of furthering the study of the equol degradation observed at the end of the kinetic experiments, some EP and NEP fecal samples were incubated in WCAB supplemented with equol, confirming in all the samples a consumption of equol that ranged between 30 and 85% after 24 h of incubation (data not shown). These results seem to indicate that a high presence of equol-degrading microorganisms could be the determining factor for NEP phenotypes, even when EP bacteria are detected in the microbiota. Therefore, the relationship between the presence of microorganisms responsible for equol production and those able to mediate equol degradation may be responsible for the ability of the microbiota to produce equol. Related to this, the consumption of beverages enriched in equol would be interesting since equol will access the plasma before reaching the colon due to its high bioavailability [41]. Nevertheless, more studies including the isolation and identification of equol-degrading bacteria should be developed in the future.

### 3.3. Effect of SB and FSB on Equol Production by S. isoflavoniconvertens

Equol production by *S. isoflavoniconvertens* was monitored through a colonic medium supplemented with SB and FSB beverages, using 5 mg/L (19.67 µM) of pure daidzein as a control (CNT). The isoflavones contained in SB were present mainly in their glycoside form, exhibiting 25.5 mg/L (61.24 µM) of daidzin, while FSB presented mainly aglycones, showing 49 mg/L (192.74 µM) of daidzein. Therefore, considering the volume of each beverage used, the fermenters supplemented with SB and FSB beverages presented concentrations of approximately 3.8 mg/L (9.13 µM) of daidzin and 7.4 mg/L (29.11 µM) of daidzein, respectively.

*S. isoflavoniconvertens* is an EP bacteria isolated from the human intestine [8,42]. It can transform aglycone daidzein into equol, via DHD, because its genome contains the genes encoding the enzymes daidzein reductase, dihydrodaidzein reductase, tetrahydrodaidzein reductase and dihydrodaidzein racemase [7,8,43,44].

**Figure 2:**
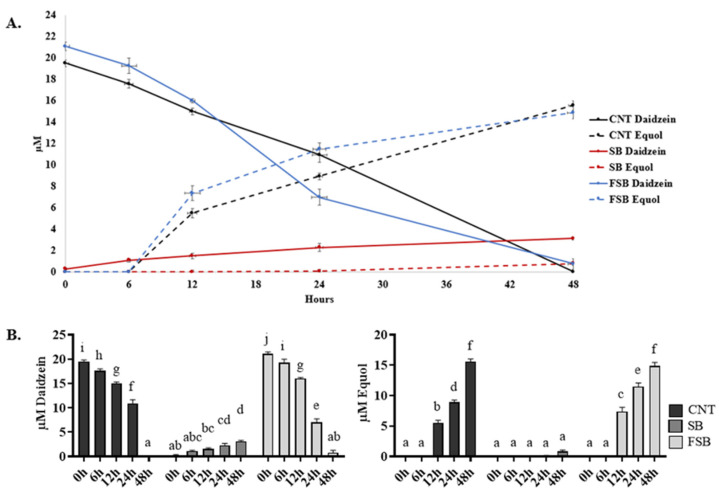
Effect of SB and FSB beverages on equol production by *S. isoflavoniconvertens*. Incubation of *S. isoflavoniconvertens* with daidzein was used as a control (CNT). (**A**) Dynamics of daidzein degradation and equol production in CNT, SB and FSB fermenters at 0, 6, 12, 24 and 48 h. (**B**) Daidzein and equol concentrations of CNT, SB and FSB fermenters at 0, 6, 12, 24 and 48 h. Values are mean ± SD. Different letters (a–j) indicate statistically significant differences (*p* < 0.05) by Tukey’s test.

In concordance with previous works, in this study, *S. isoflavoniconvertens* was able to transform the pure daidzein added to the colonic medium of the CNT fermenter into equol. After 48 h, almost all the daidzein had been metabolized and about 60% was transformed into equol (Figure 2). The formation of DHD and THD was also detected (data not shown).

Similar results were observed when *S. isoflavoniconvertens* was incubated in a colonic medium supplemented with FSB. The daidzein contained in the fermented beverage was almost completely metabolized by *S. isoflavoniconvertens* at the end of the experiment, resulting in equol (Figure 2). Equol production was first detected at 12 h in both the CNT and FSB fermenters, being significantly higher in the FSB fermenter. The maximum equol concentrations were registered at 48 h, reaching practically identical concentrations in both the CNT and FSB fermenters (Figure 2B). On the contrary, the addition of SB, which contains mainly daidzin, resulted in the production of increasing amounts of daidzein over time, which was metabolized by *S. isoflavoniconvertens* to equol, but only after 48 h of incubation (Figure 2). However, the final concentrations of daidzein and equol achieved in this fermenter were the lowest compared to those observed in the CNT and FSB fermenters. Clearly, the high concentration of daidzein present in FSB enhanced the metabolism and production of equol by *S. isoflavoniconvertens*.

Although this strain has been characterized by expressing the genes involved in the transformation of daidzein into equol [8], the results indicated that *S. isoflavoniconvertens* also exhibited a reduced glucosidase activity capable of converting daidzin to daidzein. Therefore, in order to improve this conversion of daidzin to equol, other bacteria with glycosidase activity could carry out this deglycosylation step, such as *Bifidobacterium* [43,45]. It is easy to find bacteria in the intestinal tract that carry β-glycosidases able to transform daidzin into daidzein [46]. Thus, the metabolism of isoflavones in the intestinal environment is the result of the action of a consortium of bacteria whose functions could be complementary [7,47]. However, not all individuals have the same deglycosylation and metabolization capacity, as they exhibit different intestinal microbiological profiles [7,48]. Given the complex composition of soy beverage, which contains mainly glycosylated isoflavones, a low capacity of deglycosylation by the intestinal microbiota would reduce the potential benefit of the derived compounds. Functional foods, such as fermented soy beverages, whose fermentation would be carried out with bacteria with high glycosidase activity such as *B. pseudocatenulatum* INIA P815 [24], could facilitate the biotransformation of daidzin into equol, as reflected in the results.

### 3.4. Effect of SB and FSB on the Production of Equol by EP Fecal Microbiotas

**Figure 3:**
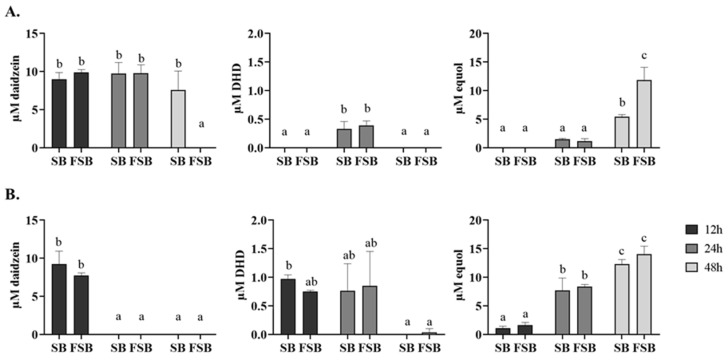
Effect of SB and FSB on equol production by EP fecal samples. (**A**) Daidzein, DHD and equol concentrations produced by FS7 after 12, 24 and 48 h. (**B**) Daidzein, DHD and equol concentrations produced by FS12 after 12, 24 and 48 h. Values are mean ± SD. Different letters (a–c) indicate statistically significant differences (*p* < 0.05) by Tukey’s test.

The EP fecal samples FS7 and FS12 were selected to evaluate their ability to produce equol through a colonic medium, based on the ability of their microbiotas to produce high concentrations of equol in a WCAB medium after 24 h (Figure 1), with FS9 being the NEP fecal sample chosen as the negative control (Table 1). Contrary to the addition of SB, the supplementation of FSB to fermenters inoculated with both the FS7 and FS12 fecal samples caused a greater impact on equol production at 48 h (Figure 3), while no production of equol was detected in fermenters inoculated with FS9 (data not shown).

Both EP fecal microbiotas FS7 and FS12 were able to deglycosylate the daidzin present in SB at similar concentrations to the daidzein exhibited in FSB after 12 h of incubation (Figure 3). However, FS7 and FS12 showed differences in their ability to metabolize daidzein into equol (Figure 3). Related to this, FS12 achieved a faster equol production rate than FS7, in agreement with the kinetic results described above (Figure 1). The fermenters supplemented with both the SB and FSB beverages incubated with FS12 exhibited daidzein, DHD, an intermediary for equol production [44] and equol after 12 h (Figure 3B). Equol production by FS12 increased over time as the precursors daidzein and DHD were consumed, regardless of the supplemented beverage in the colonic medium (Figure 3B).

Unlike FS12, FS7 required at least 24 h to produce DHD and equol, although the production rate of both compounds was low regardless of the beverage supplementation (Figure 3A).

The highest equol concentrations of the fecal microbiotas were reached at 48 h in both the SB and FSB fermenters. No significant differences in the final equol concentration were observed when FS12 was incubated in either the SB or FSB fermenters. However, although the ability of FS7 to deglycosylate daidzin allowed for the production of equol in the SB fermenter with relative efficiency, the FSB supplementation enhanced the equol production of FS7 after 48 h (Figure 3A). Moreover, the production of equol by FS7 was accompanied by the total disappearance of daidzein in the FSB fermenter. Even though FS7 showed a lower capacity to produce equol than FS12 in the SB fermenter, FSB supplementation allowed FS7 to achieve a final equol concentration similar to that observed in FS12 (Figure 3A).

As mentioned earlier, soy products such as soy beverages, contain high levels of the glycosylated isoflavones daidzin and genistin [43,49]. However, these compounds need to be bioactivated by the intestinal microbiota into their aglycones daidzein and genistein, and even better, into equol and 5-hydroxy-equol, to exert their potential health benefits [8,43]. Since the intestinal microbiota is responsible for the metabolism of isoflavones, inter-individual differences in intestinal bacterial populations would mark differences in the production and effect of the compounds [7,48]. Indeed, the gut microbial population could be classified into different isoflavone metabotypes [48]. Regarding this, although equol exerts greater effects than its precursors [7,43], only the EP individuals would take advantage of those benefits [50], even though the amount of equol produced by their intestinal microbiotas may be different among them [31,51,52,53]. Various studies indicate the importance of diet in modulating the gut microbiota [54]. In this regard, Vázquez et al. (2020) evaluated different dietary conditions to enhance endogenous equol production and revealed that a diet rich in carbohydrates enhances the synthesis of equol [52]. Other studies contemplate the production of daidzein from soy products [55] or the promotion of its biotransformation by the intestinal microbiota [46]. In previous studies, we considered the possibility of using engineered lactic acid bacteria for the development of fermented soy beverages enriched in equol and 5-hydroxy-equol, interesting for those NEP and EP individuals whose equol levels are low [43]. However, bacteria with biotechnological interest, with known qualified presumption of safety (QPS) status and the ability to produce equol in foods have not yet been described, and the use of genetically modified organisms would entail the need to eliminate the microorganisms as well as their DNA from the beverage [43,56]. Therefore, the development of soy beverages with a high concentration of daidzein either through fermentation by bacterial strains with high glycosidase activity [24] or through the application of isoflavone glycosidase enzymes [55] would be of great interest for EP individuals with a lower capacity to produce equol.

## 4. Conclusions

The incubation of the fecal samples from different volunteers with daidzein showed a proportion of EP similar to that described in the Western population. Although the presence of the *tdr* gene in most fecal samples suggests a great potential to produce equol, different underlying factors could hinder the production of this compound. In view of the results, we postulate that the degradation of equol by the microbiota could be a determining factor for the lack of production of equol by the intestinal microbiota harboring the *tdr* gene. Although the ability of intestinal microbiota to deglycosylate daidzin resulted in the production of equol from SB with low efficiency, the high concentration of daidzein provided by FSB could ensure higher production of equol in those microbiotas with elevated rates of equol degradation.

## Figures and Tables

**Table 1:** Phenolic compounds analyzed for microbial degradation of equol.

Compounds	Molecular Formula	[M − H]—(*m*/*z*)
3,4-dihydroxyphenylacetic acid	C_8_H_8_O_4_	1,670,350
4-Hydroxyphenylacetic acid	C_8_H_8_O_3_	1,510,401
3-(3,4-Dihydroxyphenyl) propionic acid	C_9_H_10_O_4_	1,810,506
2-(4-hydroxyphenyl)-propionic acid	C_9_H_10_O_3_	1,650,557
Protocatechuic acid	C_7_H_6_O_4_	1,530,193
Catechol	C_6_H_6_O_2_	1,090,295
3-(3-Hydroxy-4-methoxyphenyl) propionic acid	C_10_H_12_O_4_	1,950,663

**Table 2:** Metabolism of daidzein by adult human fecal microbiota and presence of *tdr* gene.

Fecal Samples (FS)	Equol (>1 µM)	*O*-DMA (> 1 µM)	*tdr*	Individual Characteristics
FS1	−	+	+	Female, 30–50 years
FS2	−	+	+	Female, 30–50 years
FS3	−	−	+	Female, 30–50 years
FS4	+	+	+	Female, >50 years
FS5	+	+	+	Female, <30 years
FS6	−	+	+	Female, <30 years
FS7	+	+	+	Male, 30–50 years
FS8	+	+	+	Female, <30 years
FS9	−	−	+	Male, >50 years
FS10	−	+	+	Female, >50 years
FS11	−	+	−	Female, 30–50 years
FS12	+	+	+	Male, 30–50 years
FS13	+	+	+	Male, >50 years
FS14	−	+	+	Male, >50 years
FS15	−	+	−	Male, <30 years
FS16	−	+	+	Female, 30–50 years
FS17	−	+	+	Female, >50 years

“+” indicates presence of the compound/gene, while “−” indicates absence.

## Data Availability

The authors declare that the data supporting the findings of this study are available within the article.
